# Resveratrol inhibits decidualization by accelerating downregulation of the CRABP2-RAR pathway in differentiating human endometrial stromal cells

**DOI:** 10.1038/s41419-019-1511-7

**Published:** 2019-03-20

**Authors:** Asako Ochiai, Keiji Kuroda, Rie Ozaki, Yuko Ikemoto, Keisuke Murakami, Joanne Muter, Akemi Matsumoto, Atsuo Itakura, Jan J. Brosens, Satoru Takeda

**Affiliations:** 1 Department of Obstetrics and Gynaecology, Faculty of Medicine, Juntendo University, Tokyo, 113-8421 Japan; 2 Center for Reproductive Medicine and Implantation Research, Sugiyama Clinic Shinjuku, Tokyo, 116-0023 Japan; 3 The Division of Biomedical Sciences, Clinical Science Research Laboratories, Warwick Medical School, Coventry, CV2 2DX UK; 4 Tommy’s National Centre for Miscarriage Research, University Hospitals Coventry & Warwickshire, Coventry, CV2 2DX UK

## Abstract

Pregnancy critically depends on the transformation of the human endometrium into a decidual matrix that controls embryo implantation and placenta formation, a process driven foremost by differentiation and polarization of endometrial stromal cells into mature and senescent decidual cells. Perturbations in the decidual process underpin a spectrum of prevalent reproductive disorders, including implantation failure and early pregnancy loss, emphasizing the need for new therapeutic interventions. Resveratrol is a naturally occurring polyphenol, widely used for its antioxidant and anti-inflammatory properties. Using primary human endometrial stromal cell (HESC) cultures, we demonstrate that resveratrol has anti-deciduogenic properties, repressing not only the induction of the decidual marker genes *PRL* and *IGFBP1* but also abrogating decidual senescence. Knockdown of Sirtuin 1, a histone deacetylase activated by resveratrol, restored the expression of *IGFBP1* but not the induction of *PRL* or senescence markers in decidualizing HESCs, suggesting involvement of other pathways. We demonstrate that resveratrol interferes with the reprogramming of the retinoic acid signaling pathway in decidualizing HESCs by accelerating down-regulation of cellular retinoic acid-binding protein 2 (CRABP2) and retinoic acid receptor (RAR). Notably, knockdown of CRABP2 or RAR in HESCs was sufficient to recapitulate the anti-deciduogenic effects of resveratrol. Thus, while resveratrol has been advanced as a potential fertility drug, our results indicate it may have detrimental effects on embryo implantation by interfering with decidual remodeling of the endometrium.

## Introduction

Resveratrol (3,5,4′-trihydroxystilbene), a natural polyphenolic compound found in grapes, nuts, and berries, is widely studied because of its antioxidative, anti-inflammatory and insulin-sensitizing properties^1,2^. It is a potent activator of Sirtuin 1 (SIRT1), a nicotinamide adenine dinucleotide (NAD)-dependent histone deacetylase that targets numerous transcription factors involved in metabolic homeostasis, cell differentiation, apoptosis and senescence^3–5^. Resveratrol has been mooted as a potentially therapeutic agent for infertile patients with diminished ovarian reserve, obesity, and polycystic ovary syndrome (PCOS)^6–10^. In addition, a number of studies pointed towards the therapeutic potential of resveratrol in improving testicular function and sperm quality as well as in the management of pelvic endometriosis and uterine leiomyomas^11–13^. However, the potential impact of resveratrol on endometrial preparation for embryo implantation has not yet been evaluated.

During the mid-luteal phase, the human endometrium starts to remodel intensely, heralding a transient window for embryo implantation. This implantation window coincides with the differentiation of endometrial stromal cells into specialized decidual cells. In pregnancy, tightly adherent decidual cells form a nutritive matrix around the early conceptus that controls trophoblast invasion and regulates maternal immune tolerance of the antigenically-distinct fetus^14^. A recent study demonstrated that human endometrial stromal cells (HESCs) polarize upon differentiation into mature and acutely senescent decidual subpopulations^15^. Interleukin-15 (IL-15) activated uterine natural killer (uNK) cells then target and eliminate senescent decidual cells via granule exocytosis, enabling the cycling endometrium to transition into a gestational tissue upon embryo implantation^15^.

Differentiation of HESCs into mature decidual cells critically depends on coordinated reprogramming of multiple signaling pathways^16–19^, including the retinoic acid (RA) pathway^20,21^. The cellular responses to RA are mediated by distinct nuclear receptors; RA receptors (RAR) and peroxisome proliferator activated receptors (PPAR) β/δ, which promote apoptosis and cellular differentiation, respectively^22–24^. The nature of the cellular response to RA signaling is regulated by two intracellular RA-binding proteins, cellular retinoic acid-binding protein 2 (CRABP2) and fatty acid-binding protein 5 (FABP5). RA bound to CRABP2 in the cytoplasm activates the RAR, which leads to hetero-dimerization with retinoid X receptor (RXR) and activation of genes associated with cell cycle arrest and apoptotic machinery. By contrast, binding of RA to FABP5 activates PPARβ/δ, which induces cellular differentiation. Upon decidualization, HESCs downregulate CRABP2 and, to a lesser extent, FABP5. RAR is also downregulated whereas PPARβ/δ, which transduces the differentiation responses of RA, is induced^20^.

SIRT1 is an important modulator of RA signaling. For example, SIRT1 interacts with and deacetylates CRABP2, which sequesters this RA binding protein in the cytoplasm^25^. SIRT1 also suppresses the transcriptional activity of RAR^26,27^. In light of these observations, we hypothesized that resveratrol-mediated SIRT1 activation in HESCs would favor the FABP5-PPARβ/δ pathway, thereby promoting decidualization. Unexpectedly, we observed that exposure of primary culture to resveratrol blocks subsequent differentiation of HESCs into mature and senescent cells by accelerating downregulation of CRABP2-RAR pathway.

## Results

### Resveratrol suppresses decidualization

**Fig. 1:**
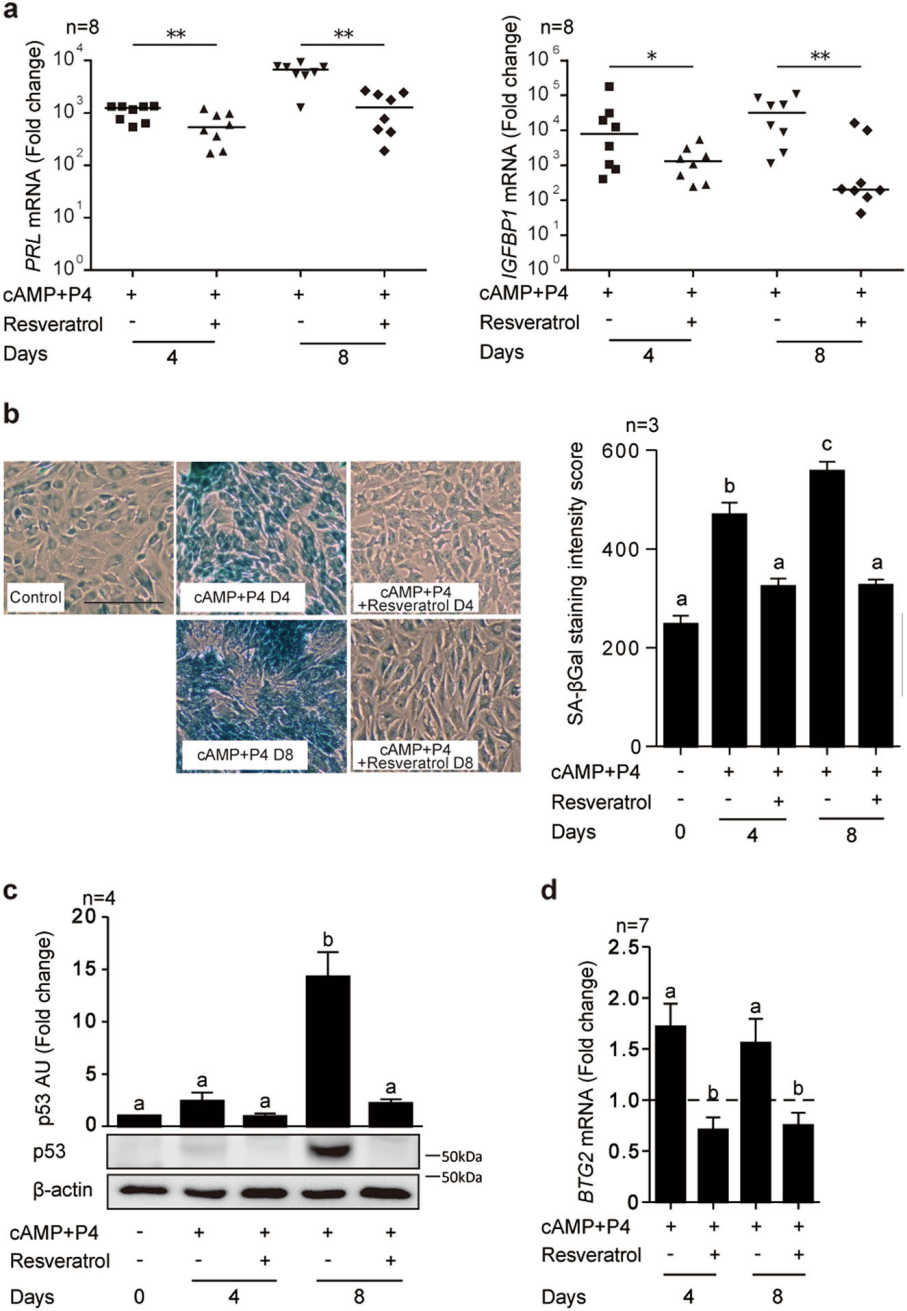
Resveratrol inhibits decidualization **a** RTQ-PCR analysis of *PRL* and *IGFBP1* transcript levels in primary HESC cultures (*n* = 8) treated with cAMP and P4 in combination with or without resveratrol (100 µM) for 4 or 8 days. *P < 0.05; **P < 0.01. **b** Representative senescence-associated β-galactosidase (SAβG) staining of parallel cultures. Original magnification: ×100. Scale bar: 200 μm (left panel). SAβG staining intensity score calculated from 300 cells in three random fields. Data show fold-change (mean ± SEM) relative to vehicle control (right panel). **c** Representative Western blot analysis and quantification of p53 levels in whole-cell lysates from 4 independent cultures decidualized in the presence or absence of resveratrol for the indicated timepoints. *β*-actin serves as a loading control. **d** RTQ-PCR analysis of *BTG2* transcript levels in primary HESCs treated as indicated (*n* = 7). Data show fold-change (mean ± SEM) relative to vehicle-treated undifferentiated cells (dotted line). Different letters above error bars denote significant difference at *P* < 0.05

An initial dose-response experiment indicated that 100 µM of resveratrol significantly inhibited the induction of *PRL* (encoding prolactin), a widely used decidual marker gene, in primary cultures treated with 8-bromoadenosine 3′5′-cyclic AMP (cAMP) and progesterone (P4) for 4 days (Supplementary Figure S1). To validate this observation, 8 independent primary cultures were decidualized with cAMP and P4 for either 4 or 8 days in the presence or absence of 100 µM of resveratrol. As shown in Fig. 1a, resveratrol not only inhibited the induction of *PRL* but also attenuated *IGFBP1* (coding insulin-like growth factor-binding protein-1) expression in decidualizing cultures. By contrast, pre-treatment of primary cultures with 100 µM resveratrol for 48 h followed by wash-off and decidualization in the absence of resveratrol had no impact on the induction of either *PRL* and *IGFBP1* (Supplementary Figure S2). Thus, the anti-deciduogenic actions of resveratrol are temporally confined to the process of active differentiation.

Recent studies demonstrated that primary HESCs polarize into two subpopulations upon decidualization, representing mature and acutely senescent decidual cells^15,28^. To examine the impact of resveratrol on decidual senescence, primary cultures differentiated for either 4 or 8 days in the presence or absence resveratrol were stained for senescence-associated β-galactosidase (SAβG) activity, a widely used biomarker for senescent cells^29^ (Fig. 1b). As expected, decidualization was associated with a time-dependent increase in SAβG^+^ cells. Resveratrol abolished the induction SAβG^+^ cells in primary HESCs treated with cAMP and P4 (Fig. 1b). To substantiate this observation, we examined the levels of the tumor suppressor protein p53, a critical mediator of decidual senescence^15,30^, in primary cultures decidualized in the presence or absence of resveratrol. Again, resveratrol abrogated p53 accumulation in differentiating HESCs (Fig. 1c). We also examined the induction of B cell translocation gene 2 (*BTG2*), an anti-proliferation gene induced by RA in MCF-7 breast cancer cells^31,32^. *BTG2* expression was upregulated upon decidualization, in keeping with the need for HESCs to exit the cell cycle prior to differentiation^15^, and again this response was blocked by resveratrol (Fig. 1d). Taken together, the data show that resveratrol is a potent inhibitor of decidualization, including decidual senescence.

### SIRT1 knockdown does not reverse resveratrol-mediated decidual repression

**Fig. 2:**
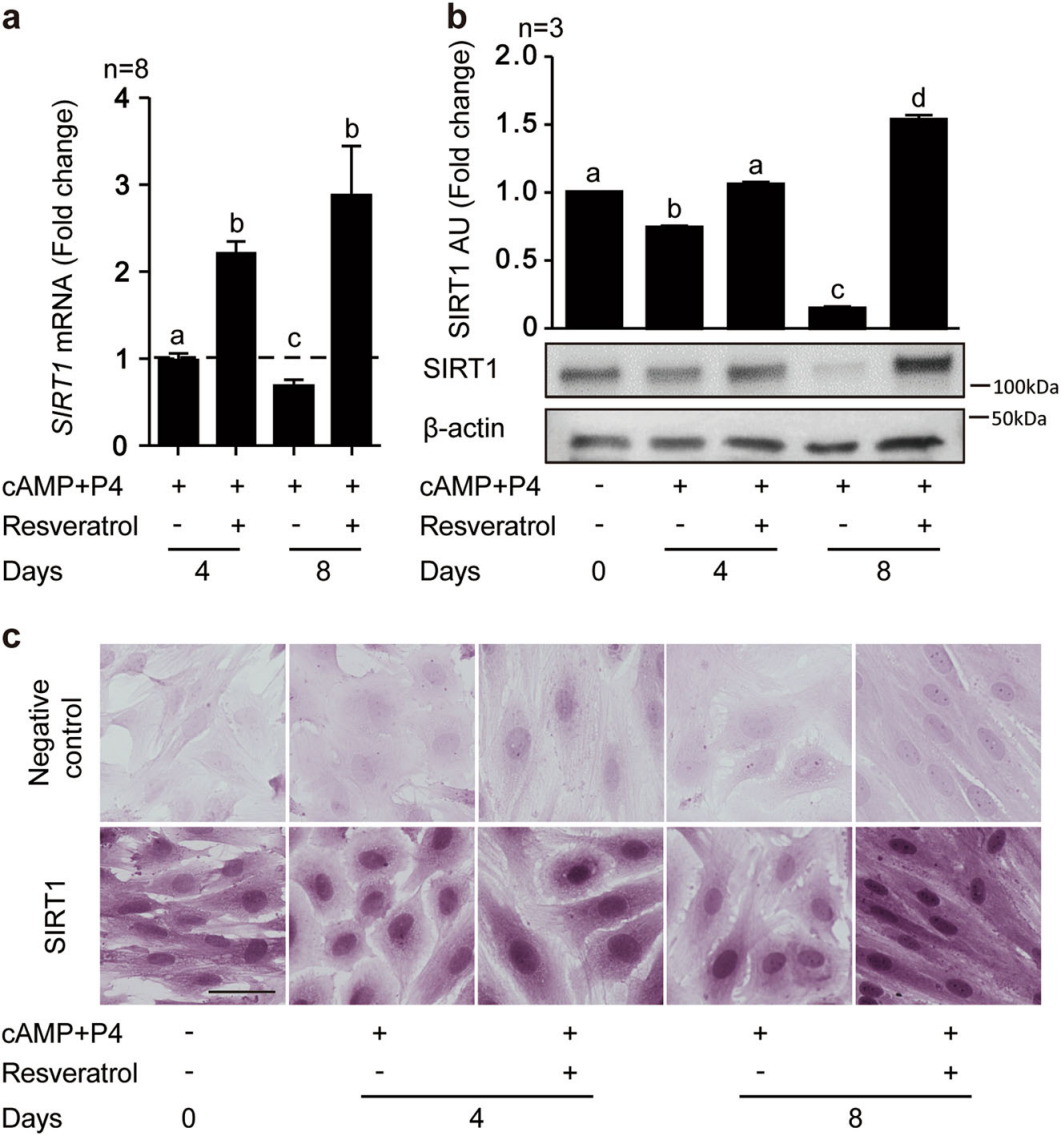
Resveratrol regulates SIRT1 expression in decidualizing HESCs. **a** *SIRT1* mRNA levels in undifferentiated and decidualized cells treated with cAMP and P4 in combination with or without resveratrol (100 µM) for 4 or 8 days. Data show fold-change (mean ± SEM) relative to undecidualized vehicle control (dotted line) of 8 independent experiments. **b** Representative Western blot and quantification of SIRT1 levels in whole cell lysates from 3 independent cultures treated in parallel. *β*-actin serves as a loading control. Different letters above error bars denote significance at *P* < 0.05. **c** Representative immunohistochemical images of SIRT1 expression in HESC cultures treated as indicated. Original magnification: ×200. Scale bar: 50 μm

To determine the mechanism of resveratrol action in differentiating HESCs, we first profiled the expression of SIRT1, a NAD-dependent deacetylase activated by resveratrol, in primary cultures differentiated for 4 and 8 days in the presence or absence of resveratrol treatment. As shown in Fig. 2a, *SIRT1* transcript levels were unchanged after 4 days of decidualization with cAMP and P4 but down-regulated by day 8. By contrast, decidualization in the presence of resveratrol resulted in significant increase in *SIRT1* mRNA levels at both time-points (*P* < 0.01). Western blot analysis showed that downregulation of SIRT1 in decidualizing cells was more pronounced at protein level when compared to mRNA level (Fig. 2b), potentially indicating a posttranscriptional mechanism of regulation. Nevertheless, resveratrol reversed the downregulation of SIRT1 in response to cAMP and P4 treatment resulted in significantly higher SIRT1 levels by day 8 of decidualization (*P* < 0.05). Immunohistochemistry confirmed nuclear accumulation of SIRT1 upon decidualization in the presence of resveratrol for 4 or 8 days. Further, acquisition of the typical decidual morphology, characterized by nuclear and cytoplasmic enlargement, was blocked in the presence of resveratrol with cells retaining a fibroblastic appearance (Fig. 2c).

**Fig. 3:**
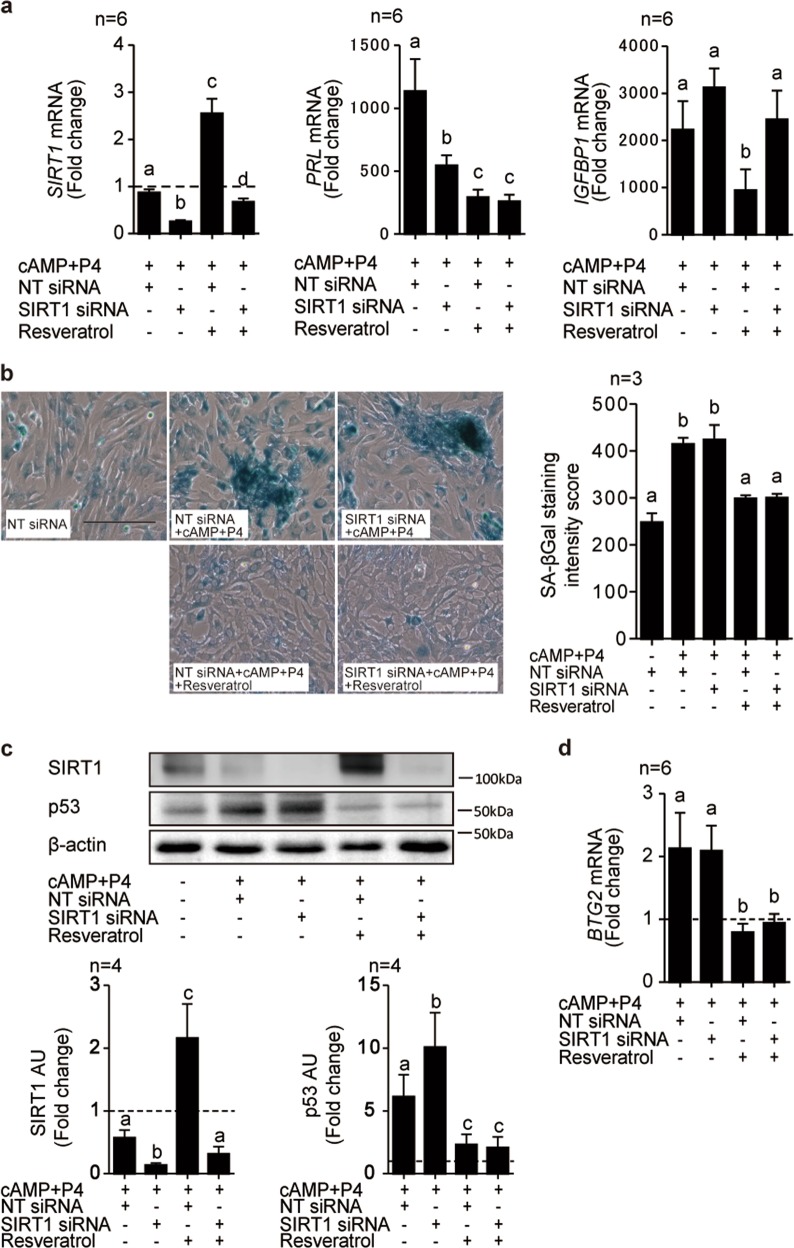
Impact of SIRT1 knockdown on resveratrol actions in decidualizing HESCs. **a** RTQ-PCR analysis of *SIRT1*, *PRL*, and *IGFBP1* transcript levels in HESC cultures first transfected with NT or SIRT1 siRNAs and then treated with cAMP and P4 in combination with or without resveratrol for 4 days. The experiment was carried out in 6 independent primary cultures. **b** Representative SAβG staining of parallel HESC cultures. Original magnification: ×100. Scale bar: 200 μm. SAβG staining intensity score was calculated from 300 cells in 3 random fields (right panel) and data show mean ± SEM score of 3 independent cultures. **c** Representative Western blot and quantification of SIRT1 and p53 proteins in whole-cell lysates obtained from parallel HESC cultures (*n* = 4). *β*-actin serves as a loading control. **d** RTQ-PCR analysis of *BTG2* transcript levels in 6 independent primary cultures treated as indicated. Data show fold-change (mean ± SEM) relative to vehicle-treated undifferentiated cells (dotted line). Different letters above error bars denote significance at *P* < 0.05

To determine if SIRT1 activation was responsible for the anti-deciduogenic actions of resveratrol, primary cultures were first transfected with either non-targeting (NT) siRNA or siRNA targeting SIRT1 and then decidualized with cAMP and P4 in the presence or absence of resveratrol for 4 days. The knockdown efficacy was high with SIRT1 mRNA level in resveratrol treated cultures remaining below that observed in untreated control cells (Fig. 3a, left panel). Interestingly, SIRT1 knockdown had no effect on resveratrol-mediated inhibition of *PRL* expression (Fig. 3a, middle panel), whereas *IGFBP1* expression was restored (Fig. 3a, right panel). Further, SIRT1 knockdown did not restore decidual senescence in resveratrol-treated cultures as ascertained by SAβG (Fig. 3b). To corroborate this observation, total protein lysates of cultures first transfected with either NT or SIRT1 siRNA and then decidualized for 4 days in the presence or absence of resveratrol were subjected to Western blot analysis. As shown in Fig. 3c, SIRT1 knockdown increased p53 accumulation in decidualizing cultures, although downregulation of p53 in response to resveratrol treatment was not reversed. Furthermore, SIRT1 knockdown was also insufficient to reverse resveratrol-mediated downregulation of *BTG2* mRNA levels in decidualizing cultures (Fig. 3d). Taken together, the data indicate that SIRT1 activation in response to resveratrol exposure of decidual cells accounts for repression of selective decidual genes, such as *IGFBP1*. However, resveratrol-mediated repression of other decidual features, including p53 accumulation and cellular senescence, are SIRT1-independent.

### Resveratrol accelerates downregulation of the CRABP2-RARα pathway in decidualizing cells

**Fig. 4:**
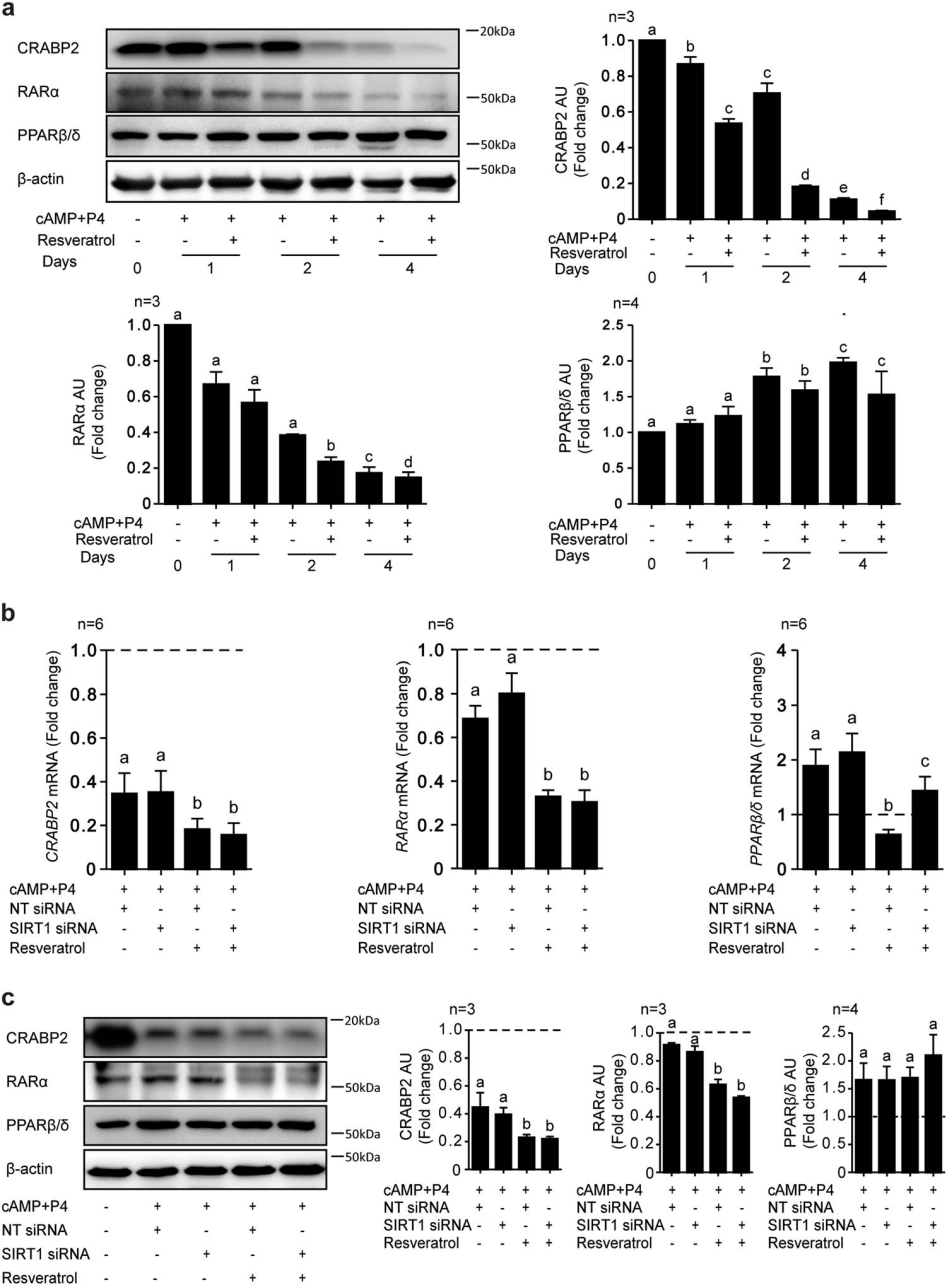
Resveratrol advances the downregulation of the CRABP2-RARα pathway in decidualizing HESCs. **a** Representative Western blot and quantification of CRABP2, RARα, and PPARβ/δ proteins obtained from whole-cell lysates in undifferentiated or decidualized cells treated with cAMP and P4 in combination with or without resveratrol (100 µM) for the indicated timepoints (*n* = 3). *β*-actin serves as a loading control. **b** RTQ-PCR analysis of *CRABP2*, *RARα*, and *PPARβ/δ* transcript levels in HESC cultures (*n* = 6) first transfected with NT or SIRT1 siRNA and then treated with cAMP and P4 in combination with or without resveratrol for 4 days. **c** Representative Western blot and quantification of RA related genes in whole cell lysates (*n* = 3) from parallel primary cultures. *β*-actin serves as a loading control. Data show fold-change (mean ± SEM) relative to vehicle-treated undifferentiated cells (dotted line). Different letters above error bars denote significance at *P* < 0.05

In other cell systems, resveratrol has been shown to modulate a number of signal transduction pathways, including RA signaling^26,33^. Decidual transformation of HESCs is accompanied with suppression of CRABP2-RARα signaling and induction of PPARβ/δ, involved in cellular differentiation^20^. To monitor the impact of resveratrol on this pathway, total protein lysates from primary HESC cultures, decidualized for 1, 2, or 4 days in the presence or absence of resveratrol, were harvested for Western blot analysis. As expected, differentiation of HESCs with cAMP and P4 resulted in a time-dependent downregulation of CRABP2 and RARα expression and concomitant rise in PPARβ/δ levels (Fig. 4a). Interestingly, resveratrol attenuated the induction of PPARβ/δ and accelerated the downregulation CRABP2 and RARα, a response that was already apparent after 24 h of decidualization and maintained over 4 days.

Next, we performed additional real-time quantitative (RTQ)-PCR and Western blot analyses to determine if the effect of resveratrol of RA signal transduction pathway was SIRT1-dependent. As shown in Fig. 4b, c, SIRT1 knockdown had no impact on CRABP2 and RARα expression in decidualizing cells, irrespectively of resveratrol treatment. By contrast, SIRT1 knockdown was sufficient to reverse resveratrol-mediated inhibition of PPARβ/δ at both mRNA and protein level. The data further confirm that resveratrol impacts on decidual gene expression through SIRT1-dependent and SIRT1-independent mechanism.

### Accelerated downregulation of the CRABP2-RARα pathway impairs decidualization

**Fig. 5:**
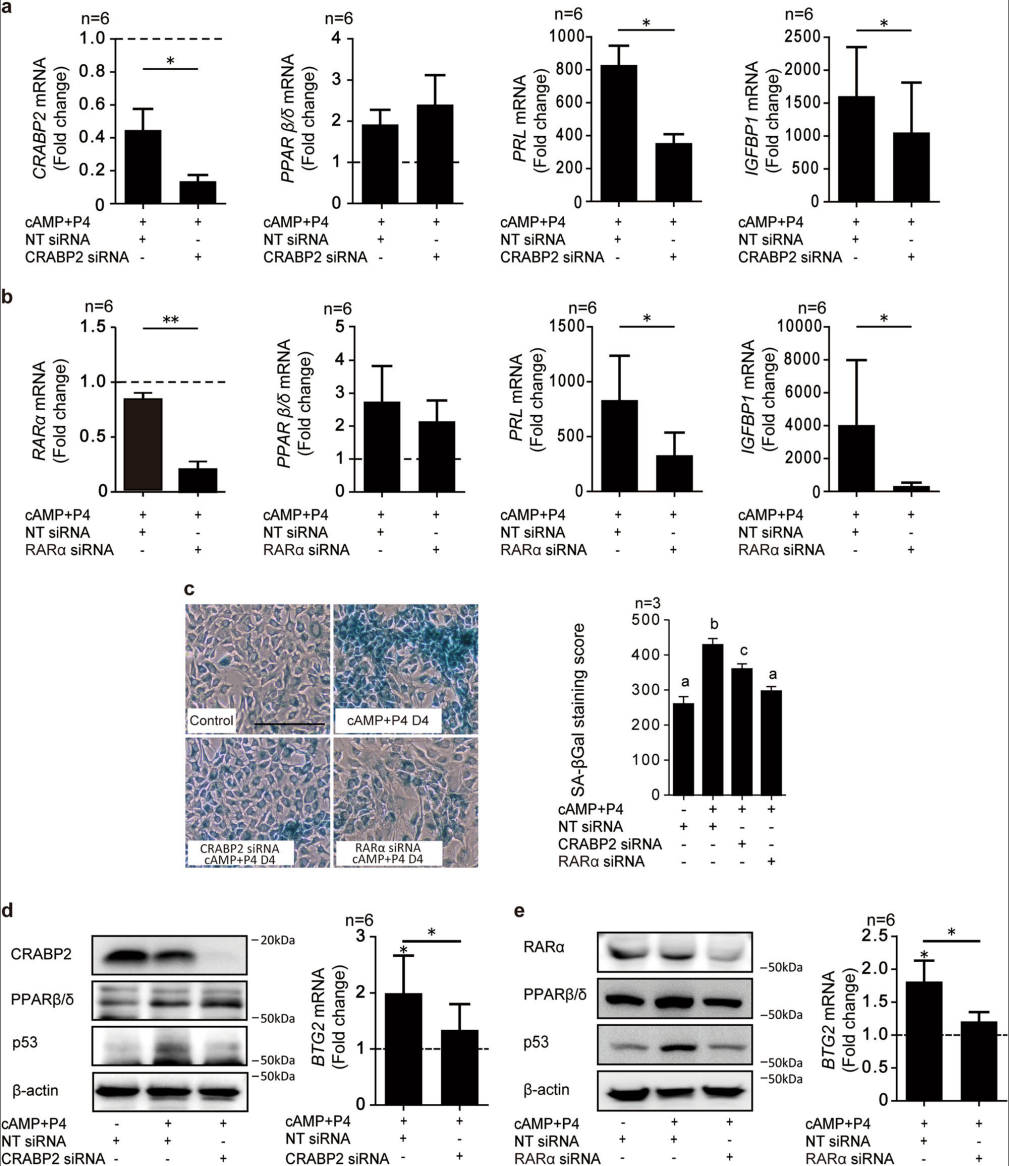
CRABP2 or RARα knockdown inhibits decidualization. **a, b** RTQ-PCR analysis of *CRABP2*, *RARα*, *PPARβ/δ*, *PRL*, and *IGFBP1* transcript levels in 6 independent HESC cultures first transfected with NT, CRABP2 (**a**) or RARα (**b**) siRNA and then treated with cAMP and P4 for 4 days. Data show fold-change (mean ± SEM) relative to vehicle control (dotted line). *P < 0.05; **P < 0.01. **c** Representative SAβG staining of 3 independent HESC cultures first transfected with NT, CRABP2, or RARα siRNAs and then decidualized for 4 days. Original magnification: ×100. Scale bar: 200 μm. Data shows SAβG staining intensity score resulted from 300 decidual cells in 3 random fields Different letters above error bars denote significance at *P* < 0.05 (right panel). **d**, **e** Representative Western blot of RA-related gene proteins and p53 in whole-cell lysates obtained from 6 independent HESC cultures treated as indicated. *β*-actin serves as a loading control (left panel). RTQ-PCR analysis of *BTG2* transcript levels in primary parallel cultures (right panel). The results show the fold-change (mean ± SEM)

Previous studies reported that differentiation of HESCs into decidual cells is a multi-step process, dependent on coordinated down- and up-regulation of distinct gene networks^17,18,21^. Hence, we speculated that accelerated downregulation of the CRABP2-RARα pathway in response to resveratrol may be sufficient to disrupt the subsequent decidual phenotype. To test this hypothesis, three independent primary HESCs were first transfected with NT siRNA or siRNA targeting CRABP2 or RARα and then decidualized with cAMP and P4 (Fig. 5a, b). When compared to undifferentiated cells, *CRABP2* mRNA levels were reduced by 60 and 90% in cells decidualized for 4 days following transfected with NT or CRABP2 siRNA, respectively. CRABP2 knockdown had no impact on PPARβ/δ expression but repressed the induction of *PRL* and *IGFBP1* in decidualizing cells (Fig. 5a). Knockdown of RARα recapitulated these findings (Fig. 5b). Notably, GW501516, a PPARβ/δ agonist, further enhanced the expression of its target nuclear receptors in cells decidualized for 4 days, but had no impact on either *PRL* or *IGFBP1* mRNA levels (Supplementary Figure S3). Taken together, the data indicate that accelerated downregulation of the CRABP2-RARα pathway blunts the induction of decidual marker genes in HESCs.

Finally, we investigated if coordinated downregulation of the CRABP2-RARα pathway in differentiating HESCs is required for the emergence of senescent decidual cells. As shown in Fig. 5c, knockdown of CRABP2 or RARα was sufficient to attenuate the induction of SAβG in primary cultures treated with cAMP and P4 for 4 days. As expected, knockdown of CRABP2 or RARα also inhibited p53 accumulation and induction of *BTG2* expression in decidual cells (Fig. 5d, e).

## Discussion

A limited window of implantation synchronizes embryo implantation in an optimal uterine environment. In mice, a postovulatory oestradiol surge both activates dormant embryos and renders the progesterone-primed endometrium receptive^34^. Recent evidence suggests that an acute auto-inflammatory endometrial response has replaced the obligatory maternal implantation signal in mice^15^. In human endometrium, this inflammatory response associated with the window of implantation is thought to polarize HESCs into two decidual subpopulations^15^, through an evolutionarily conserved mechanism^35^. The first subpopulation consists of mature decidual cells, which will form a semi-permanent, nutritive matrix that controls trophoblast invasion and placental formation in pregnancy. The second subpopulation, however, are HESCs that failed to acquire a specialized phenotype and emerge as acutely senescent cells that secrete various pro-inflammatory cytokines, chemokines and proteases involved in tissue remodeling. Targeting and selective clearance of acutely senescent decidual cells by uNK cells terminates the inflammatory implantation window and effectively rejuvenates the endometrium at the time of embryo implantation^15^. An aberrant decidual response is associated implantation failure, recurrent pregnancy loss and preeclampsia^36–39^. Preventing these prevalent reproductive disorders is a clinical priority but challenging because of a lack of effective therapeutic interventions.

Resveratrol is a widely used and well tolerated compound exhibiting anticancer^40^ immunomodulatory^41^ and antioxidant properties^42,43^. Although increasingly advocated for the treatment of reproductive failure associated with PCOS and obesity^8–10,44^, we demonstrate here that resveratrol is a potent inhibitor of the decidual pathway. We previously reported that differentiating HESCs downregulate CRABP2 and RAR, effectively silencing the pro-apoptotic arm of RA signaling, whilst simultaneously up-regulating the pro-differentiation RA-responsive nuclear receptor PPARβ/δ^20^. We now show that resveratrol both accelerates the downregulation of CRABP2 and RARα and supresses PPARβ/δ induction. Knockdown experiments demonstrated that premature loss of CRABP2 or RARα is sufficient to block transcriptional reprogramming of HESCs as well as the emergence of distinct decidual subpopulations. As a direct RAR target gene, BTG2 transcriptionally reduces cyclin D1 availability, inhibiting G_1_-S progression^31,45,46^. Exit from the cell cycle is a prerequisite for the emergence of terminally differentiated as well as senescent cells, and hence, indispensable for optimal decidualization of HESCs. We reported previously that cAMP-dependent downregulation of MDM2 proto-oncogene, an E3 ubiquitin ligase, stabilizes p53 in differentiating HESCs^30^. We now show that resveratrol prevents p53 accumulation in response to cAMP and P4 signaling in a SIRT-1 independent manner. It has previously been reported that resveratrol ameliorates age-related metabolic disorders by competitively inhibiting cAMP phosphodiesterases, resulting in enhanced cAMP levels^47^. Combined with a previous observation that p53 accumulates in resveratrol treated breast cancer cell lines^48^, our findings reinforce that resveratrol acts in both a tissue- and context-specific manner. Notably, p53 activates dehydrogenase/reductase 3 (DHRS3), a key enzyme in retinoid metabolism^49,50^. DHRS3 facilitates the reduction of retinal to retinol, and is induced upon decidualization^21^. As such, resveratrol-mediated suppression of p53 is likely to perturb the cellular concentrations of bioactive retinoids.

Mature decidual cells are programmed to endure a range of environmental stressors, giving rise to a protective matrix able to withstand the intense tissue remodeling and vascular changes associated with implantation and placental formation. Coordinated silencing of multiple pathways including inositol triphosphate (IP3) signaling^19^, c-Jun N-terminal kinase (JNK) signaling^17^ as well as cessation of endometrial circadian rhythms^18^ underpins this quasi-autonomous decidual state. Inhibition of the pro-apoptotic CRABP2-RAR arm of RA signaling renders the endometrium relatively resistant to the potentially adverse effects of RA. By contrast, upregulation of PPARβ/δ is a hallmark of implantation sites in the rat uterus^51^. Although. co-treatment of HESCs with a PPPARβ/δ agonist had no effect on the induction of *PRL* or *IGFBP1*, the expression levels of both genes may already be maximal in cultures treated with cAMP and P4.

In summary, resveratrol is a promising therapeutic agent in reproductive medicine^6,9,44,52^, although its impact on embryo implantation or early pregnancy loss has not yet been evaluated. Pre-treatment of primary HESC cultures with resveratrol for 48 h prior to decidualization had no detrimental effects on the induction of *PRL* and *IGFBP1*. The half-life of resveratrol is only 9–10 h in humans^53,54^. As such, limiting the use of resveratrol to the proliferative phase of the cycle may be the preferred strategy to improve ovarian function in selected patients without adversely impacting on subsequent embryo implantation, although this assertion requires further testing in clinical studies.

## Materials and methods

### Patient selection

This study was approved by the local ethical committee in Juntendo University, Faculty of Medicine (No. 14-103). Endometrial biopsies were obtained during the luteal phase of the cycle from patients without overt uterine pathology attending the Department of Obstetrics and Gynecology of Juntendo University Hospital, Tokyo, Japan. None of the participants were on hormonal therapy or taking resveratrol supplementation. Written, informed consent was obtained from all participating women prior to the biopsy.

### Primary culture of human endometrial stromal cells

Collected HESCs were isolated, cultured, and maintained as described previously^20,55^. We obtained the endometrial samples in Dulbecco’s modified Eagle’s medium-Ham’s (DMEM) F-12 (Nacalai Tesque, Kyoto, Japan) containing 1% (v/v) antibiotic solution, minced finely and digested enzymatically with 5 mg collagenase (5 mg/100 μl) (Sigma-Aldrich, Saint Louis, USA) and deoxyribonuclease (DNase) type I (100 μg/μl) (Roche Applied Science, Mannheim, Germany) for 1 h at 37 °C. After centrifugation, the cells were suspended in DMEM/F12 culture media containing 10% (v/v) dextran-coated charcoal (DCC) treated fetal bovine serum, 1% antibiotic solution, 1% (v/v) l-glutamine, 0.2% (v/v) insulin and 1 nM estradiol (all Sigma-Aldrich). Endometrial cells were cultured until confluence in 75 cm^2^ culture flasks at 37 °C in 5% carbon dioxide and then passaged once or twice. Cells were discarded after passage 3. In decidualization experiments, confluent monolayers were maintained in DMEM/F12 without phenol red (GIBCO, life technologies, Grand Island, USA) containing 2% (v/v) DCC-FBS and treated with 0.5 mM 8-bromo-cAMP, 1 μM P4 in combination with or without 100 µM resveratrol (all Sigma-Aldrich).

### Immunohistochemical staining

Confluent HESC cultures in 4-well chamber slides were decidualized as described above in the presence or absence of 100 µM resveratrol for 4 or 8 days. Following treatment, cells were fixed with 4% buffered paraformaldehyde, endogenous peroxidase activity blocked with 1% H_2_O_2_ and probed with anti-SIRT1 antibody at a 1:100 dilution overnight (Abcam, Cambridge, UK). Excess primary antibody was washed with TBS-T and probed with a secondary anti-rabbit antibody at a 1:300 dilution (DAKO, Glostrup, Denmark). The expression of SIRT1 was detected using horseradish peroxidase-conjugated streptavidin, 1:300 (DAKO) and visualized with SIGMA*FAST*™ DAB with Metal Enhancer (Sigma-Aldrich), in which Cobalt chloride was added to enhance 3,3′-diaminobenzidine reaction. Eosin was used as a cytoplasmic counterstain. Images were taken using Keyence BZ-X700 microscope with 40×, 100×, or 200× magnifications (Keyence, Osaka, Japan).

### RTQ-PCR

Total RNA was extracted from primary HESC cultures using RNeasy plus mini kit (QIAGEN, Hilden, Germany). We generated cDNA with the SuperScript II Reverse Transcriptase for RT-PCR kit (Invirtrogen Ltd. Life technology, Carlsbad, USA) and performed Template quantification using 7500 fast real-time PCR system (Applied Biosystems, Forster City, USA) with dye layer, power SYBER Green PCR Master Mix (Applied Biosystems). RNA input variances were normalized against the levels of housekeeping gene, *L19* which encodes a ribosomal protein. All measurements were performed in duplicate or triplicate. The gene-specific primer pairs were designed by using Primer3 software (http://frodo.wi.mit.edu): *L19* sense,5′-GCG GAA GGG TAC AGC CAA T-3′, *L19* antisense, 5′-GCA GCC GGC GCA AA-3′; *SIRT1* sense 5′-TCT AAC TGG AGC TGG GGT G-3′ and *SIRT1* antisense, 5′-AAG TCT ACA GCA AGG CGA GC-3′; decidual *PRL* sense 5′-AAG CTG TAG AGA TTG AGG AGC AAA C-3′ and decidual *PRL* antisense, 5′-TCA GGA TGA ACC TGG CTG ACT A-3′; IGF-binding protein 1 (*IGFBP1*) sense, 5′-CGA AGG CTC TCC ATG TCA CCA-3′ and *IGFBP1* antisense, 5′-TGT CTC CTG TGC CTT GGC TAA AC-3′; *CRABP2* sense, 5′-TGT GAG CAG AAG CTC CTG AAG-3′and *CRABP2* antisense 5′-GTT CTA CCT GTG GCC ACT CAC T-3′; *RARα* sense, 5′-GCC CAG CTC ACC ACA TCT TC-3′ and *RARα* antisense, 5′-GGA GCA ATG GCT TGT GAG TTCT-3′; *PPARβ/δ* sense, 5′-ACT GAC CCA ACT GAT CCT GCT C-3′ and *PPARβ/δ* antisense, 5′-GCC TGG CAA ACC AGT GTG AA-3′; *BTG2* sense, 5′-ACG GGA AGG GAA CCG ACA T-3′and *BTG2* antisense 5′-CAG TGG TGT TTG TAG TGC TCT G-3′. Proprietary commercially available primers for *FABP5* (QIAGEN, Maryland, USA) were used.

### Western blot analysis

Total protein lysates were extracted from HESCs by direct lysis in SDS sample buffer heated to 75 °C. Proteins resolved by SDS-PAGE were separated in a polyacrylamide gel (Bio-rad laboratories, Hercules, USA) and transferred to Immobilon-P membrane (Merck, Darmstadt, Germany) and probed with antibodies raised against SIRT1, 1:500 (Abcam) CRABP2, 1:5000 (Sigma-Aldrich); FABP5, 1:1000 (Abcam); RARα, 1:5000 (Abcam); PPARβ/δ, 1:1000 (Abcam); p53, 1:1000 (Cell Signaling Technology, Danvers, USA), and β-actin, 1:5000 (Sigma-Aldrich). After incubation with peroxidase-conjugated secondary antibodies, (Jackson Immunoresearch Laboratories, West Grobe, USA), detection of chemiluminescence was visualized with Pierce Western Blotting Substrate Plus (Thermo Fisher Scientific, Rockford, USA) or Super Signal West Dura Extended Duration Substrate (Thermo Fisher Scientific) and normalized to β-actin. Western blots were quantified by densitometry using Multi Gauge software Version3.0 (Fuji Photo Film, Tokyo, Japan).

### RNA interference

Primary HESCs were seeded in six-well plates and cultured to 70% confluence for 24 h. Cells were then transfected with siRNAs against genes encoding *SIRT1* (M-003540-0010, siGENOME SMARTpool siRNA, Dharmacon, MA, USA), *CRABP2* (M-003448-01, siGENOME SMARTpool siRNA, Dharmacon), *RARα* (M-003437-02, siGENOME SMARTpool siRNA, Dharmacon), or negative control siRNA (452001, Stealth RNAi Negative Control, invitrogen, Carlsbad, USA) using Lipofectamine RNAiMAX Reagent (invitrogen, Carlsbad, USA), according to the manufacturer’s instruction. For transfection, 25 pmol of siRNAs and 5 µl of Lipofectamine RNAiMAX were diluted with 500 µl of reduced serum medium (Opti-MEM, Invitrogen, Life Technologies) and mixed. The transfection mixture was incubated for 20 min at room temperature and added dropwise to each well containing 2 ml of DMEM/F12 with 5% (v/v) DCC-FBS (final siRNA concentration: 10 nM). Twenty-four hours following transfection, cells were washed with PBS and decidualized as described above.

### SAβG staining

Following 4 or 8 days of decidualization, cells were stained with X-gal using Senescence β-Galactosidase Staining Kit (Cell Signaling Technology, Danvers, USA) according to manufacturer’s instruction. After 24 h of incubation, cells were observed using a phase-contrast microscopy. Images were taken using Keyence BZ-X710 microscope with 40×, 100×, or 200× magnifications (Keyence, Osaka, Japan). Senescent cells were detected as blue-stained cells, the staining intensity of positive cells were scored as 0 (absent staining); 1 (partial cytoplasmic staining); 2 (total cytoplasmic staining). A total of 300 cells were counted in three random fields on a culture plate for each sample (Supplementary Figure S4).

### Statistical analysis

Data are representative of three or more biological replicates. Results are reported as mean ± standard error (SEM). Statistical analysis was performed using one-way analysis of variance (ANOVA) followed by Wilcoxon signed rank test or Mann–Whitney test within groups, following normalization of the data with GraphPad Prism 5 (GraphPad Software Inc., San Diego, USA). The level of significance was defined as *P* value of <0.05.

## Supplementary information

Supplementary Figure S1

Supplementary Figure S2

Supplementary Figure S3

Supplementary Figure S4

Supplementary figure legends
