# Supplementary figures and images for: Resveratrol inhibits decidualization by accelerating downregulation of the CRABP2-RAR pathway in differentiating human endometrial stromal cells
Source: Cell Death Dis. 2019 Mar 20;10(4):276. doi: 10.1038/s41419-019-1511-7 (PMC6427032; doi:10.1038/s41419-019-1511-7)

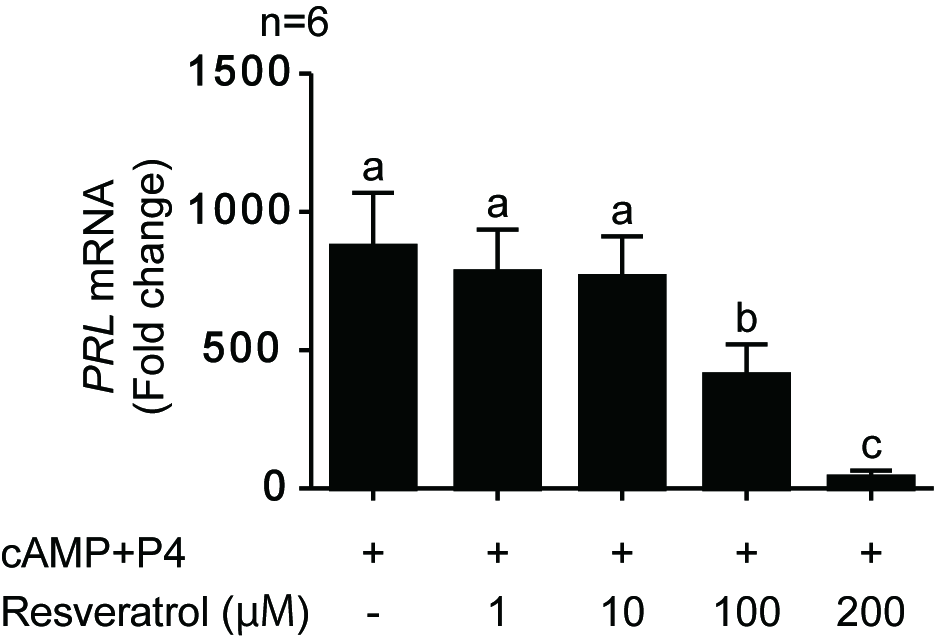

Supplement: Supplementary file 1 — Supplementary Figure S1 [file 41419_2019_1511_MOESM1_ESM.tif]

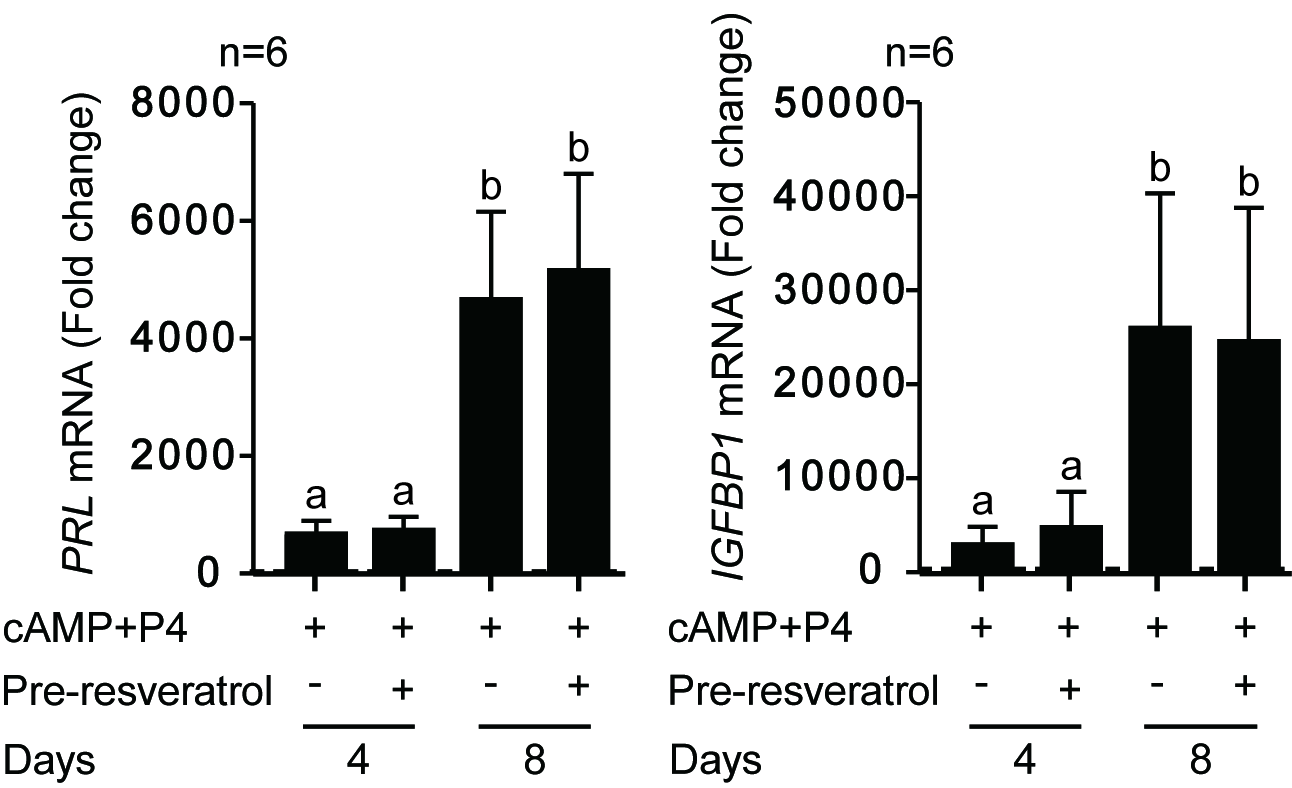

Supplement: Supplementary file 2 — Supplementary Figure S2 [file 41419_2019_1511_MOESM2_ESM.tif]

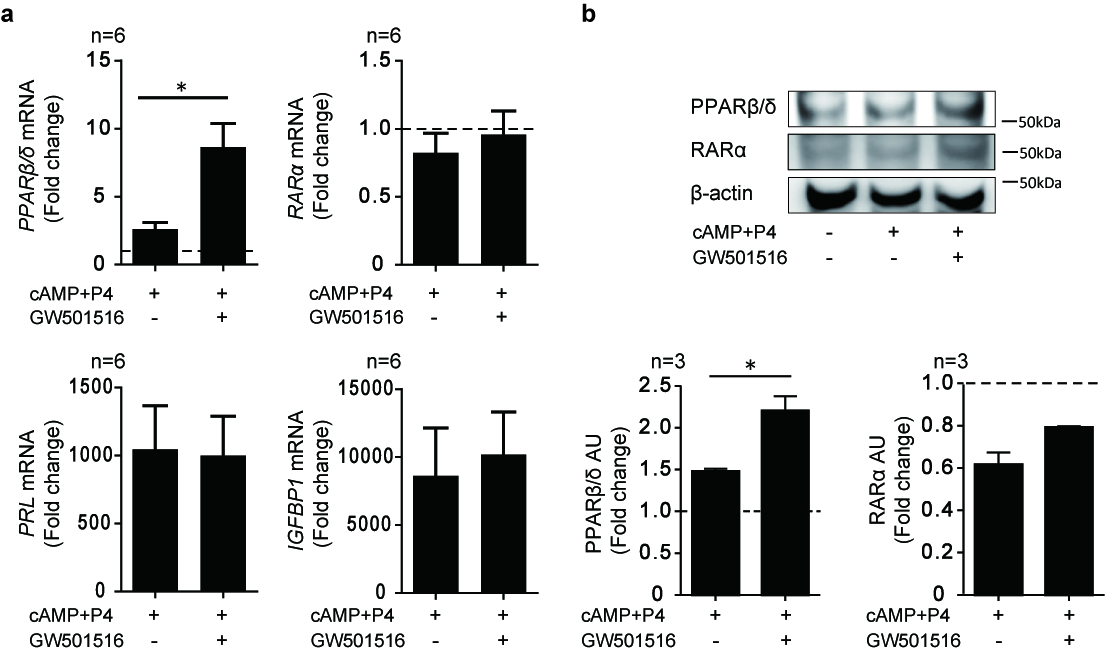

Supplement: Supplementary file 3 — Supplementary Figure S3 [file 41419_2019_1511_MOESM3_ESM.tif]

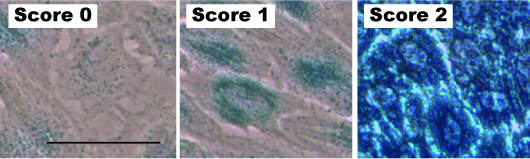

Supplement: Supplementary file 4 — Supplementary Figure S4 [file 41419_2019_1511_MOESM4_ESM.tif]
