# Supplementary material for: Resveratrol inhibits decidualization by accelerating downregulation of the CRABP2-RAR pathway in differentiating human endometrial stromal cells
Source: Cell Death Dis. 2019 Mar 20;10(4):276. doi: 10.1038/s41419-019-1511-7 (PMC6427032; doi:10.1038/s41419-019-1511-7)
Supplement: Supplementary file 5 — Supplementary figure legends [file 41419_2019_1511_MOESM5_ESM.docx]

**Supplementary Figure Legends**

**Supplementary Figure S1. Resveratrol inhibits decidual PRL expression in a dose-dependent manner.** Primary HESC cultures (n=6) were decidualized for 4 days in the presence of increasing concentrations of resveratrol as indicated. Induction of PRL expression was measured **by** RTQ-PCR analysis and date presented as fold-change (mean ± SEM) relative expression level in vehicle-treated control cells. Different letters above error bars denote significance at *P* < 0.05.

**Supplementary Figure S2. Resveratrol pre-treatment does not impair subsequent decidualization.** RTQ-PCR analysis of *PRL* and *IGFBP1* transcript levels in HESC cultures first treated with resveratrol (100 µM) for 48 hours and then treated with cAMP and P4 for 4 or 8 days. The data show fold-change (mean ± SEM) relative to vehicle control. Different letters above error bars denote significance at *P* < 0.05.

**Supplementary Figure S3. Effect of PPARβ/δ agonist, GW501516, on decidualization.** (**a**) *PPARβ/δ*, *RARα*, *PRL* and *IGFBP1* mRNA levels in undifferentiated HESCs and cells decidualized with cAMP and P4 in the presence or absence of GW501516 (10 nM) for 4 days. The data show fold-change (mean ± SEM) relative to vehicle control (*dotted line*). *, *P* < 0.05; **, *P* < 0.01. (**b**) Representative Western blot and quantification of PPARβ/δ and RARα protein in whole cell lysates from parallel cultures. *β*-actin serves as a loading control.

**Supplementary Figure S4. SAβG staining intensity scor**e. Senescent cells were identified as blue-stained cells, the staining intensity of positive cells were scored as 0: absent staining; 1: partial cytoplasmic staining; or 2: total cytoplasmic staining. A total of 300 cells were counted in three random fields per culture. Original magnification: ×200. Scale bar: 50 μm*.*
